# *‘I’ve never heard of pulmonary rehab’:* Healthcare professionals’ perceptions in regards to chronic obstructive pulmonary disease

**DOI:** 10.1177/02692155251387316

**Published:** 2025-10-22

**Authors:** Emma Swift, Mary R O’Brien, Sarah Peters, Carol Kelly

**Affiliations:** 1 School of Health and Society, 7046 University of Salford, Salford, UK; 2 Faculty of Health, Social Care and Medicine, 6249 Edge Hill University, Ormskirk, UK; 3 Manchester Centre for Health Psychology, University of Manchester, Manchester, UK

**Keywords:** Healthcare professional, pulmonary rehabilitation, COPD, exercise, psychology, education

## Abstract

**Objective:**

To identify healthcare professionals’ perceptions of pulmonary rehabilitation as a management strategy for people with chronic obstructive pulmonary disease (COPD).

**Design:**

A qualitative interview study which adopted an interpretive phenomenological approach.

**Methods:**

Twenty-seven healthcare professionals were recruited from general practices in the North West of England and two hospital trusts, consisting of: general practitioners, practice nurses, and doctors and nurses working on general medical wards. Audio recorded semi-structured interviews investigated healthcare professionals’ perceptions and knowledge of pulmonary rehabilitation; interpretive phenomenological analysis was conducted on the transcribed interviews.

**Results:**

Three themes were identified: *COPD Illness Perceptions*, *Pulmonary Rehabilitation Beliefs*, and *Organisational and Referral Pathway Perceptions*. Commonalities and disparities were identified between primary and secondary care and amongst the different professional groups. Healthcare professionals held negative COPD illness perceptions including stigmatising beliefs in relation to the disease. These beliefs impacted their referral practice. Beliefs about pulmonary rehabilitation included views about patient suitability for the pulmonary rehabilitation programme. A lack of knowledge of pulmonary rehabilitation and the referral process was evident. Surprisingly, many working on general medical wards had not heard of pulmonary rehabilitation and none in their current role had referred to the programme. Organisational and referral pathway perceptions revealed barriers and facilitators to referral.

**Conclusion:**

Referral to pulmonary rehabilitation programmes is influenced by healthcare professionals’ perceptions and knowledge of pulmonary rehabilitation, referral pathways and how COPD affects patients. Together health professional perceptions could act as a predictor of referral practice and inform strategies for increasing referral rates.

## Introduction

Pulmonary rehabilitation is an established cost-effective evidenced-based intervention which reduces chronic obstructive pulmonary disease (COPD) related hospital admissions.^1^ A non-pharmacological therapeutic management strategy, pulmonary rehabilitation is delivered by a multidisciplinary team, providing individualised care for patients regardless of where they are on the disease trajectory.^2^ The aim is to assist with COPD self-management by offering support in three domains: physical, social and psychological.^3^ Pulmonary rehabilitation should therefore be multifaceted and encompass an exercise regime to increase activity, education, breathing techniques, medication advice and psychological support.^4,5^

Regardless of the programme's proven effectiveness, attendance and adherence for patients with COPD is low.^6^ Access, participation and attrition is a global issue, speculatively due to a lack of availability of the programme in low- or middle-income countries and being underutilised or inadequately resourced in high-income countries.^5^ Literature highlights the perceived barriers to accessing pulmonary rehabilitation for individuals with COPD.^7^ Some refuse referral believing their condition is self-inflicted due to smoking^8^; others feel anxious, believing exercise induces breathlessness, a feeling they wish to avoid.^9^ Transportation issues are also highlighted,^6^ in addition to pulmonary rehabilitation often taking place during working hours.^8^ Many are unfortunately unaware of pulmonary rehabilitation and cannot recall being offered referral.^10^ Factors relating to income and location, which contribute to overall socio-economic status can also influence adherence.^11^

It is recommended pulmonary rehabilitation should be provided to all who meet the criteria defined in COPD guidelines^12^ along with those admitted to hospital with an exacerbation of their COPD.^4^ However, the findings from The National Asthma and COPD Pulmonary Rehabilitation Audit^13^ highlight only 5.2% (632) of UK patients were referred to pulmonary rehabilitation after admission to hospital with an acute exacerbation of COPD. Thus, improvement in access is required in secondary care, in addition to primary care referrals.

Currently, literature exists surrounding patients’ perceptions of pulmonary rehabilitation,^14^ however little is known regarding healthcare professionals’ views. A systematic review drew attention to healthcare professionals’ perceptions with a significant focus upon the barriers to pulmonary rehabilitation, such as a lack of healthcare professional knowledge, diminished time to make a referral, and practical barriers such as transportation and long waiting lists.^15^ Most referrals made to pulmonary rehabilitation in the UK are from primary rather than secondary care,^13^ although those referred by a general practitioner (GP) are less likely to complete the programme.^16^ Predictors of referral in primary care in Wales highlight it is less likely for those who are a current smoker, over the age of 70, female, deprived, or have a comorbidity such as asthma or a condition which leads to pain.^17^ It is evident healthcare professionals are not referring patients as frequently as they could, however the reasons for this are unclear.^18^ Much of the current literature regarding healthcare professionals’ perceptions focusses upon those with the ability to refer in primary care, with limited understanding of whether the perceptions of those in secondary care are similar.^15^ The current study therefore aims to identify the perceptions of healthcare professionals both in primary and secondary care regarding pulmonary rehabilitation for patients with COPD.

### Objectives

1. To establish healthcare professionals’ understanding of pulmonary rehabilitation.
2. To explore the perceptions that healthcare professionals, both in primary and secondary care, have about referring those with COPD to pulmonary rehabilitation.
3. To explore barriers and facilitators to referral to pulmonary rehabilitation.

## Methods

University ethical approval was obtained, in addition to National Health Service (NHS) Health Research Authority approval (IRAS ID: 208153). Purposeful recruitment of healthcare professionals with the ability to refer COPD patients to pulmonary rehabilitation was employed. Ability to refer to pulmonary rehabilitation was the homogenous factor. Participants were recruited from four professional groups: GPs and practice nurses in primary care, and doctors, and nurses working on general medical wards in secondary care. Recruitment took place in two clinical commissioning groups, and two hospital trusts in the North West of England. A general medical ward was defined as a ward with no specialism, which cared for patients with various conditions. This included, but was not exclusive to, medical assessment units, GP assessment areas, ambulatory units, general medicine departments, elderly care wards, acute medicine, and clinical decisions wards. A letter with a reply slip was sent to those working in primary care, and an email was sent by a gatekeeper to eligible healthcare professionals working on general medical wards in secondary care. A second strategy, due to a low response rate in secondary care, enabled the lead researcher (ES) to accompany the gatekeeper to general medical wards to discuss details of the study in person with the healthcare professionals working there.

### Data collection

Individual semi-structured interviews were conducted; congruent with standard data collection methods for interpretive phenomenological analysis (IPA) studies.^19^ All interviews were digitally audio recorded with the participants’ consent and took place at a mutually convenient time. Interviews conducted in primary care (*n* = 14) were all via telephone, at participants’ request. Conversely, most interviews in secondary care (*n* = 11) were conducted face to face at the hospital where the healthcare professional worked. These typically took place in offices off the general medical ward, or in an unoccupied family or day room. The remainder of secondary care interviews (*n* = 2) were conducted via telephone. Questions focussed on: experience with respiratory conditions, understanding and perceptions of pulmonary rehabilitation, the referral process, factors influencing a referral decision, and if they had received any feedback on the programme and its effectiveness. Details of participant demographics are provided in Tables 1 and 2.

**Table 1.** Participant demographics primary care.

Participant ID	Age	Interest	List size
GP 1	61 +	General practice	<5000
GP 2	31–40	Women's health	5001–7000
GP 3	41–50	General medicine, often works with respiratory patients with multiple chronic conditions	13,001–16,000
GP 4	31–40	Palliative care	13,001–16,000
GP 5	41–50	Respiratory medicine, research and epidemiology	7001–10,000
GP 6	51–60	General practice	10,001–13,000
GP 7	41–50	Children's, women's health and dermatology	7001–10,000
GP 8	51–60	Musculoskeletal, women's health and diabetes	7001–10,000
PN 1	61+	General practice	7001–10,000
PN 2	41–50	General practice	16,001 +
PN 3	51–60	Respiratory conditions	10,001–13,000
PN 4	51–60	General medicine	10,001–13,000
PN 5	25–30	Diabetes	7001–10,000
PN 6	41–50	Respiratory conditions	10,001–13,000

GP: General practitioner; PN: Practice nurse.

**Table 2.** Participant demographics secondary care.

Participant ID and role	Age	Interests	Hospital site	Ward type
DR 1 Junior Doctor (FY1)	25–30	Surgery	Hospital 1	Acute Medical Unit
DR 2 Junior Doctor (FY1)	25–30	Cardiology	Hospital 1	Acute Medical Unit/Endocrine
DR 3 Registrar	31–40	A & E	Hospital 1	Acute Medical Unit/A & E
DR 4 Registrar	25–30	General medicine	Hospital 1	Acute Medical Unit
DR 5 Junior Doctor (FY2)	25–30	Anaesthetics	Hospital 2	Acute Medicine Unit
DR 6 Junior Doctor (FY1)	25–30	General practice	Hospital 2	Acute Medicine Unit/Ambulatory Emergency Care Unit
GN 1 Nurse	51–60	Intensive care and acute medicine	Hospital 2	Acute Medicine Unit
GN 2 Nurse	41–50	Acute medicine	Hospital 1	Acute Medical Unit
GN 3 Nurse	20–25	Acute medicine	Hospital 1	Acute Medical Unit
GN 4 Nurse	41–50	Acute medicine	Hospital 2	Assessment Unit
GN 5 Nurse	51–60	Elderly Care	Hospital 2	Acute frailty unit/Assessment and Rehabilitation Day Unit
GN 6 Nurse	41–50	General medical	Hospital 2	Acute frailty unit/Assessment and Rehabilitation Day Unit
GN 7 Nurse	41–50	Respiratory and acute medicine	Hospital 2	Acute Medical Unit/Ambulatory Emergency Care Unit

DR: Doctor working on a general medical ward; GN: Nurse working on a general medical ward; FY1: Foundation doctor (year one); FY2: Foundation doctor (year two).

A researcher reflexive diary was completed during the recruitment process by ES, with notes being taken after each interview, transcription, and during data analysis. This process allowed for any initial thoughts, feelings, or interpretations to be accounted for and later incorporated into the analysis.^20^

### Data analysis

**Figure 1.**
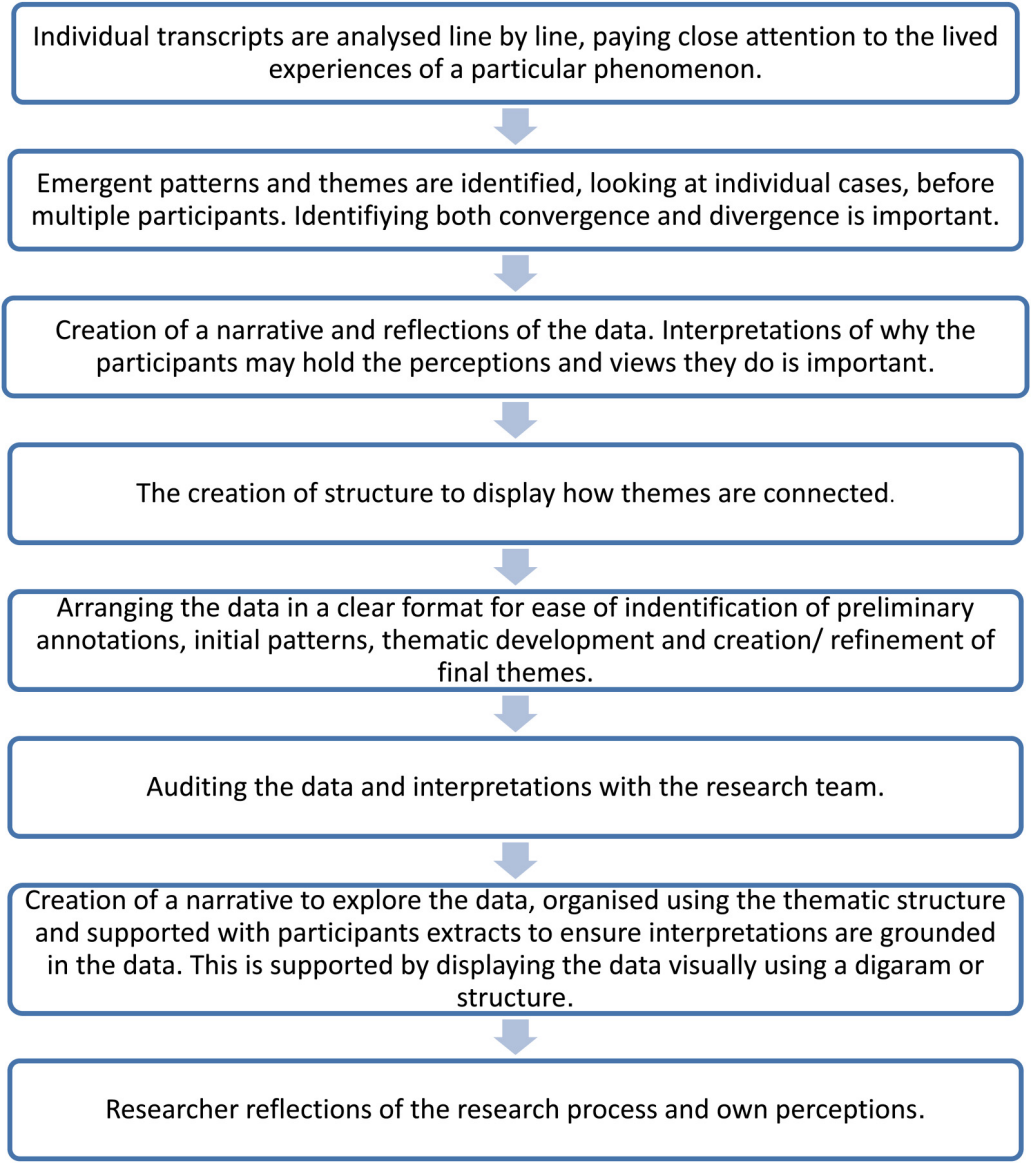
The main steps of IPA data analysis (adapted from Smith et al., 2009).^20^

IPA was employed. Verbatim transcription occurred after each interview. Commencing this process prior to the next interview allowed for instant immersion in the data and for any interesting new topics or emergent themes to be noted and incorporated into subsequent interviews.^19^ Analysis was conducted using NVivo 11^®^ and due to the ideographic nature of IPA^20^ transcripts were analysed case by case and line by line, with notes made of any pertinent quotes in relation to healthcare professionals’ perceptions of pulmonary rehabilitation. As greater familiarity with the transcript was achieved, similarities, disparities and contradictions in participant perceptions throughout the transcript were noted.^20^ The process of IPA has been summarised in Figure 1.

The data were analysed by ES and audited by CK to ensure super-ordinate and sub-ordinate themes were grounded in the data; agreement was achieved. A narrative was formed with the intention to take the reader on a journey through the participants’ experiences and views, providing verbatim quotes, discussing interpretation and acknowledging instances of disparity.^20^

## Results

Twenty-seven participants, consisting of GPs (*n* = 8) and Practice Nurses (*n* = 6) from primary care, and Doctors (*n* = 6) and General Nurses (*n* = 7) from secondary care, were interviewed. Table 3 details individual participant characteristics and general perceptions of pulmonary rehabilitation; this has been displayed to preserve the ideographic nature of IPA and allow the reader to become familiar with each participant before reading the narrative.

**Table 3.** Individual participant characteristics and general perceptions of PR.

Participant:	How Often they Refer Patients to PR:	General Perceptions of PR:
Primary Care:		
GP 1	‘O*n average every two months or so’*. Deferred responsibility believed that it was the practice nurses role during COPD annual reviews.	Tentative over potential benefits of PR; perceived it could give patients the ‘*confidence to undertake exercise*’ and an ‘*understanding of their condition’*. Stated many benefits would be ‘*non- specific’* and held limited local programme knowledge, especially surrounding eligibility criteria, believing PR is most suitable for patients at the ‘*worse end of the spectrum’*.
GP 2	Infrequently: Believed this was mainly the practice nurse's role.	Believed PR was for those who lacked *‘knowledge’* of COPD and were ‘*not responding to treatment*’. Local programme knowledge was ‘*limited’*, however appreciated PR would help educate patients, as those delivering the programme have ‘*more time’*. Did not ‘*prioritise’* PR due to time restraints or because ‘*you just don’t think about it at the time’*.
GP 3	Does not refer: ‘*other people tend to be doing that other than me’*	Knew ‘*very little’* about PR. Perceived it was to help patients ‘*live with their condition’*, however was unconfident when discussing the programme and often related it to pain management, which she appeared more familiar with. Appeared unconvinced of its benefits as patients ‘*often tell me they haven’t found it useful*’.
GP 4	Never referred: Defers responsibility to the respiratory department in secondary care.	Believed PR was an ‘*exercise programme*’ to improve ‘*breathing*’ of COPD patients. She was aware that she could refer patients but ‘*did not get too involved*’. Lacked local programme knowledge and was unaware the practice nurses could refer. Admitted she wanted to take part to raise awareness there are HCPs with very little knowledge of PR.
GP 5	Infrequently: Only ‘*new patients’* who are ‘*MRC 3 and below’*. Possibly four or five in the practice per year.	Believed PR was ‘*individually set’* for patients and extremely useful for breathlessness. Perceived it had ‘*more beneficial effects than most of the inhaled medication*s’; it also aids psychologically, reducing ‘*social isolation’*. Had some local programme knowledge yet was unaware of how often patients attended PR. Referral did not appear prioritised as he added that he hoped he ‘*remembered*’ to refer.
GP 6	Does not refer: Places referral responsibility on the practice nurse, to complete during annual COPD review.	Aware of PR and associated benefits. Perceived it can offer as much ‘*relief as even the inhaler based therapies*’. Understood the referral criteria, however had ‘*limited*’ local programme knowledge. Considered it difficult to convince patients the programme will be ‘*beneficial*’, and felt there was a lack of contact and information from the service.
GP 7	Never referred: Uncertain GPs can refer patients to PR.	Unsure of local programme specifics, however understood the programme involves education, ‘*exercises’* and believed there were ‘*some games’*. Lacked knowledge of eligibility criteria and the benefits. Considers waiting lists may be long and wanted more information.
GP 8	Previously referred: Now considers it the nurse practitioners’ role in the COPD annual review.	Knowledgeable about the local programme and defined PR as helping patients to ‘*develop exercise tolerance’*, ‘*educate’* and assist with ‘*breathing exercises’*. Appreciated the benefits, however viewed that it is ‘*frustrating’*, that many patients do not attend. Perceived there was limited information available and felt the information provided by the local service online was tailored to the elderly.
PN 1	Frequently refers: ‘*it happens every week’*	Passionate about PR, assists with all aspects of the disease, rather than just medication. Perceived it helps psychologically, increasing ‘*confidence’* and socialisation; viewed the ‘*light exercise’* and education increases ‘*quality of life’*. She was pro-active and visited the local programme to increase her knowledge, to allow her to give the patients an ‘*idea of what to expect’*. Positively promotes PR to all patients, however annoyed and felt de-valued by the service over the ‘*one way road of information’*. She stated that better communication was required providing feedback on patient outcomes and details of local programme specifics.
PN 2	Refers: ‘*maybe once every three months’*	Knowledgeable and passionate about how PR can change patients’ lives. Perceived the programme involves ‘*exercise*’ and ‘*education*’ and is delivered by a ‘*respiratory nurse*’ or *physio therapis*t’. Believed it is particularly beneficial for the psychological aspects and ‘*anxiety*’ associated with COPD. Perceived a lack of consistency with the local service and issues with funding have led to deterioration, which has caused a difficult and changeable referral process. Due to the referral process being difficult she perceived she was too busy to refer some patients, and was also uncomfortable with the lack of local programme knowledge, therefore she felt unable to fully inform patients.
PN 3	Frequently tries to refer: ‘*I consider everyone that hasn’t been on it’*	Knowledgeable and extremely passionate about local programme and ‘*discusses it with everyone*’; believes ‘*it is more important than half the inhalers’*. Perceived patients complete ‘*physical work under supervision of the physio, they then have a coffee and a chat*’ before ‘*education*’. Believed it was challenging to refer patients in the area she currently works, and feels responsible when ‘*99 percent’* of COPD patients attended at her previous surgery. She attributed this to her patients being younger, and that it is a ‘*high cannabis use area’*, and perceived it difficult for them to understand ‘*something that isn’t medicine is going to help*’.
PN 4	Refers: ‘*one or two a month’*	Aware PR is recommended for COPD patients and believed the programme educates patients about their condition; to ‘*manage the impact it has on their life’*. Lacks local programme knowledge, believes that the service is changeable, with differing locations. Views the support and contact for ongoing needs, that patients receive when attending PR is beneficial, along with meeting those ‘*suffering the same condition’*. Perceived it difficult to get patients to attend, and may increase anxiety seeing those in a worse position than themselves.
PN 5	Now refers frequently: Just been made responsible for the COPD patients at the surgery ‘*due to staff changes’*	Believed that PR is ‘*cost effective*’ and that ‘*patients who go have great outcomes’*. Perceived it involves exercise and education, and that it is beneficial for ‘*patients to meet other people with the condition*’. Was uncertain about the location of the programme and exactly which HCPs delivered it. Believed that patients either ‘loved’ or ‘*absolutely hated*’ it. Was positive with regards to PR increasing patient knowledge, and viewed that some particularly benefitted from attendance. Considered that transport and language barriers were issues, along with it being a difficult sell for those who considered themselves an ‘*expert patient*’.
PN 6	Refers based on patient suitability: May refer a number of patients in a short space of time and then none for a while, as the nurse practitioner and community matron also refer.	Very knowledgeable about PR and the referral criteria, and tried to sell it in a ‘*very positive manner’*. Her enthusiasm stemmed from seeing the benefits of the programme first-hand. Described how when patients return to the surgery after PR, they are aware of suitable exercises, have greater ‘confidence’ and ‘*seem to have more energy’*. Perceived that there was no disadvantages to the actual programme, as there was even transport provided. However, believed a patient barrier after referral was the wait to commence as it was ‘*months and months*’. Disappointed over the lack of ‘*feedback*’ from the service, and believed that it would be helpful to receive a ‘*brief letter’* detailing patient improvement.
Secondary Care:		
DR 1	‘Never referred’: Unaware of the referral process.	Text-book understanding of PR, believed that it was a non-pharmacological management strategy which incorporated ‘*exercise*’, to ‘*improve breathing’* and quality of life. Previously completed a placement on the respiratory wards, where patients *‘seemed quite engaged’* with PR. Perceived that patients do need to take ownership of their health and want to attend PR for themselves. Viewed there would be ‘*logistical issues’* associated with transportation, and difficulties for patients who require ‘*oxygen’*. Lacked knowledge of referral criteria and the local programme; would ‘*consider’* referral if the service provided more information. However, did also defer responsibility to primary care as she considered it a ‘*holistic approach*’.
DR 2	Never referred: ‘*I didn’t really know it was a service’*	No knowledge of PR, suggests that it may be a similar concept to cardiac rehabilitation, which she was knowledgeable about. Due to inadequate knowledge she was unaware and unconvinced of the benefits, however ‘*assumed’* that PR would have an evidence base, otherwise the NHS would not support it. Acknowledged a lack of confidence with respiratory conditions and deferred responsibility to the respiratory team. Was ‘*not averse’* to PR and ‘*would refer’* if supplied with more information from the service.
DR 3	Never referred: ‘*brand new to me’*	Inadequate knowledge, presumed that PR may be ‘*something like a chest physio’*. Was provided with a brief definition of the programme, and some questions were answered based upon her perceptions of the definition given. Perceived that it may help to reduce hospital admissions, educate patients on the correct time to present at hospital, and allow them to carry out self-management. Unaware of how to refer, however would only initiate a referral on recommendation of a consultant. Deferred responsibility to the respiratory team as she does not ‘*diagnose COPD’*. Sees COPD patients frequently, although they had not mentioned attendance at PR.
DR 4	Never referred: Did not consider it his role.	A good general understanding of PR. Believed the programme was usually ‘*led by respiratory physiotherapists*’ and focussed on ‘*reconditioning*’, via exercise and education. Perceived it would be beneficial in allowing patients to gain independence; assisting psychologically and aiding ‘*mobility’*, thus promoting ‘*survival*’. Also knowledgeable about the evidence base. Viewed that the disadvantages of PR would be that some patients may ‘*find it too difficult*’ and discussed the ‘*inverse care law*’, suggesting the patients who need PR most, probably cannot access the programme. Previously, had completed a four month respiratory placement, and currently sees COPD patients frequently. Perceived it was not his role to refer and deferred responsibility to other healthcare professionals. Unaware of the referral process, and considered programme knowledge needs to be raised.
DR 5	Never referred: ‘*I don’t know about pulmonary rehabilitation’*	Lack of awareness of PR. Admitted that it was his second day working in the Acute Medical Unit (AMU), however ‘*had lots of exposure*’ to COPD, as it is something that he sees every day. Misinterpreted the purpose of PR and stated they had a hospital discharge team, and asked if they had the same role. Was provided with a definition of PR, due to a dearth of knowledge. After the definition, he perceived PR would be useful to educate patients, however viewed that an issue may be encountered due to COPD patients lacking ‘*compliance*’. Believed there was a lack of focus on the programme during his medical degree, however learned about other services such as stroke rehabilitation; suggested the service needs to be advertised better.
DR 6	Never referred: ‘*I’d probably have to speak to a respiratory doctor to find out how to refer’*	Believed PR is for patents with ‘*quite severe COPD*’. Had good knowledge of the key components of PR, and aware that it encompassed physical and psychological support ‘to improve their exercise tolerance and independence’. Gained his knowledge sitting in COPD clinics, whist on a specially selected placement during his medical training; he was newly qualified working on AMU as his ‘*first job as a doctor*’. Perceived he had enough knowledge to discuss PR with patients, but would be unable to make the referral without assistance. A barrier to making a referral, would be a patient not willing to ‘*comply*’. Reluctant about the overall benefits ‘*it can be quite useful*’, and perceived that awareness needs to be increased amongst ‘*general medical doctors’*.
GN 1	Never referred: Not considered her role and held negative perceptions.	Very limited knowledge of PR, emailed prior to the interview to ask if there was anything she needed to revise; appeared uncomfortable over her lack of awareness. Presumed the programme should encompass: psychological support, coping mechanisms, and enable patients to remain in the community. Believed there is no cure, and therefore ‘*not a medical problem that you can actually deal with*’. Deferred responsibility of referral throughout the interview, and did not perceive it her role to assist with the management of COPD; viewed it was her job to provide support for the ‘*acute issue’*. She had spoken to another nurse on the ward prior to the interview, who had previously worked in the community. This nurse described ‘*how every time people went into pulmonary rehab, they would exacerbate and end up coming into hospital’*. She now held the same negative perceptions.
GN 2	Never referred: ‘*It's not something that I’ve heard of at all’*	Total lack of programme knowledge; aware of cardiac rehabilitation but not PR. Deferred responsibility of referral to the discharge team, ‘*who are called in at the last minute*’ to assist, however was unsure of their role. Unaware of referral criteria, and overall appeared to have a lack of interest in the programme and COPD management. Interview raised her knowledge of the programme.
GN 3	Never referred: Not heard of PR prior to the interview.	Complete lack of awareness of PR, and lacked knowledge of what the programme involves. Highlighted that during her nursing degree she ‘*might have been taught about it but I can’t remember anything’*. Unaware of the referral criteria, however perceived that PR may be something that the discharge team at the hospital deliver, believing ‘*maybe they do pulmonary rehab*’. Deferred responsibility, highlighting that patients on AMU ‘*are really poorly’*, therefore referral may be something other wards would consider, if patients are transferred. Highlighted, if she was to consider referral she would require a simplified system, with support and reassurance to decrease responsibility. Overall, would like to receive more information from the service.
GN 4	Never referred: ‘*I’ve never had to refer anyone to it, I ‘ve never been asked to either’*	Uncertainty surrounding PR, asked ‘i*s it about when they are going home’*. Participant was provided with a definition to clarify, and responses to questions were based upon this. Discussed how her mum had been diagnosed with COPD and assumed she would have attended PR, however ‘*could not manage*’ it. Frequently saw COPD patients on the ward, and viewed PR could assist with anxiety and breathing techniques, however perceived patients would need to be motivated. Did not view referral to the programme as her role, although towards the end of the interview started to consider the benefits, describing the programme as a ‘*safety net*’. Highlighted, she would lack confidence in making the referral, however if this could be sent electronically with an expert making the final decision on patient suitability, ‘*more people would probably do it*’.
GN 5	Never referred: Unfamiliar with PR, misinterpretation of the word ‘*rehab*’.	Complete lack of knowledge of PR, and seemed to become anxious when asked questions surrounding COPD and the programme. A definition of PR was provided, however he lacked enthusiasm and appeared uninterested in who referred patients to the programme. The term PR appeared to cause confusion, as he discussed how he previously worked on the ‘*rehab ward that was for hips*’. Frequently interrupted and talked over questions about topics he had not been asked; this was perceived as a result of having no knowledge. Throughout the interview defended his belief that it was not his role to refer patients. At the end apologised for knowing very little, but stated that this was because ‘*it's not our area*’. Did however believe that anything which would reduce COPD admissions, would be beneficial.
GN 6	Never referred: ‘*I wasn’t aware I could’*	Belief that PR incorporates exercises, breathing techniques and ‘*monitoring*’. Lacked clarity around programme delivery. Perceived PR would be beneficial to increase exercise tolerance, and promoted the psychological benefits. Also considered it may help to reduce loneliness and seclusion, and provide a sense of togetherness, especially for those on oxygen. Prior experience ‘*years ago*’ of working on a respiratory ward, however was unaware the programme was something available, which she could refer to. Very enthusiastic about the programme ‘*it sounds fab’*. Blamed a lack of publicity from the service for her lack of awareness. Keen to increase knowledge and asked many questions following the interview. Said she would contact the respiratory team for further details on how to refer.
GN 7	Previously referred patients ‘*all of the time’* when working in primary care: Not referred anyone since working in secondary care	When working in primary care championed PR and ‘*used to advocate it for everyone*’. Was extremely knowledgeable, and aware of the programme benefits, evidence base and COPD guidelines. Believed that it helps to: ‘*educate*’, giving patients a realistic overview of their condition, ‘*pre-empt and reduce exacerbations’*, provide peer support, and enables self-management; improving quality of life. However, perceived some patients do not want to attend because ‘*they get stuck in their ways’*. Considered her current job is to get patients ‘*over the acute phase*’ and not to refer to PR, as she believed someone else would do this. Did state she would like more local programme knowledge, as she believed things will have changed since working in primary care. Realised that as she sees COPD patients ‘*every day’*, she has an influential position, and should possibly refer.

The data have been organised and displayed under three super-ordinate themes: COPD Illness Perceptions, Pulmonary Rehabilitation Beliefs, and Organisational and Referral Pathway Perceptions. Details of the different sub-ordinate themes within each super-ordinate theme for primary and secondary care can be seen within Table 4. Within the analysis each quote will be followed by a participant identifier in brackets to acknowledge which participant perception is being discussed. For example: (GN 2, 41–50, H2). GN represents general nurse, then follows the age range of the participant and H2 depicts the hospital the participant was recruited from.

**Table 4.** Super-ordinate and sub-ordinate themes.

Super-ordinate Themes	Sub-ordinate Themes Primary Care	Sub-ordinate Themes Secondary Care
COPD Illness Perceptions	The psychological impact of COPD	Perceived patient burden
	Adds pressure to the NHS	Adds pressure to the NHS
	Stereotypical beliefs surrounding COPD	Stereotypical beliefs surrounding COPD
Pulmonary Rehabilitation Beliefs	Beliefs of what PR entails and patient suitability	Perceived patient suitability for PR
	Uncertainty	'So what is it?’
	It's helpful	Appreciation of potential benefits
	Perceived barriers to PR	Perceived barriers to PR
Organisational and Referral Pathway Perceptions	Defers responsibility	Defers responsibility
	Lack of information from the service	Lack of awareness and publicity
	Difficult referral	Unaware of patient suitability and how to refer
	Facilitators to referral	

### COPD illness perceptions – primary care

In primary care illness perceptions were evident, with many healthcare professionals having a strong belief about the psychological impact which a diagnosis of COPD can have. There was a perceived negative influence of COPD on both emotional and mental health:

‘what we have to remember is living with a life-long illness requires a lot of sort of emotion, and mental resources’ (PN 3, 51–60)

It was deemed many with COPD are anxious, and this impinged patients’ lives; the worry and apprehension can alter belief in abilities and cause refrainment from activities. As a result, individuals enter a ‘*breathlessness vicious cycle*’ (PN 5, 25–30), whereby they become trapped in a continuum of inactivity, due to trepidation surrounding perceived shortness of breath. GPs discussed how those with COPD wanted a quick fix, a ‘*miracle answer to everything*’ (GP 3, 41–50). They viewed this group as resistant to change, particularly surrounding exercise, with the perception that medical intervention was only wanted via prescription:

‘it's you know exercise, it can’t possibly make them significantly better, and it has to be something on prescription, you know be it a tablet or an inhaler, and why could anything else work.’ (GP 6, 51–60)

Many held similar stereotypical beliefs and drew attention to the perceived stubbornness and unwillingness of those with COPD. It was a struggle to convince patients that exercise was appropriate, with them often creating excuses rather than being open-minded:

‘they’ve often got an answer for it. If you tell them to go swimming, “oh I don’t like swimming, I can’t swim, it's too cold the water”. They’ll come out with everything, rather than realising that you know walking, swimming, whatever, is going to improve their health in the long run.’ (GP 8, 51–60)

There was a consistent belief that ‘*COPD has been caused by smoking’* (GP 7, 41–50*)*, and this was defined as the root cause for the disease by those working in primary care. It was felt that COPD was more commonly being diagnosed at a younger age, with one GP associating the disease with those living in a socially deprived area and being in receipt of state benefits:

‘We’ve got people in their forties… it's quite a deprived big council estate, most of them on benefits and they’re all still smoking’ (GP 8, 51–60)

### COPD illness perceptions – secondary care

Within secondary care healthcare professionals perceived COPD as a burden for individuals and a ‘*deteriorating condition’* (GN 2, 41–50, H2). General health and symptoms were sensed to deteriorate as the disease progresses. It was deemed a particularly troublesome condition with an inability to restore health to its previous state:

‘it just tends to basically get worse … you can’t cure it as such’ (GN 1, 51–60, H2)

Shortness of breath was considered a demanding aspect. Many doctors associated breathlessness with their medical knowledge of the mechanics of ventilation. Although knowledgeable about symptoms, definitions significantly focussed on the multi-facetted, unpredictable nature, its impact upon breathing and limits on exercise:

‘it's a chronic condition, associated with sort of fixed airway obstruction and difficulty with ventilating, patients tend to have sort of exertional shortness of breath, chronic cough, often they produce a lot of sputum’. (DR 4, 25–30, H1)

Those in secondary care perceived COPD added pressure to the NHS and this was commonly associated with COPD patients being viewed as frequent attenders at hospital. COPD was noted as ‘*probably one of the most* [common] *things we tend to see*’ (GN 1, 51–60, H2). Although sometimes a primary presentation, many discussed how patients on general medical wards regularly had COPD in ‘*the background’* (DR 4, 25–30, H1). The condition was believed to trigger a cyclical process, whereby an individual becomes better whilst in hospital, but shortly after discharge they become unwell, and require readmittance:

‘we see a lot of exacerbations of COPD and they’re multiple admittances’ (GN 5, 51–60, H2)

Those in secondary care also held stereotypical COPD illness beliefs, perceiving COPD was ‘*most commonly caused by smoking’* (DR 6, 25–30, H2). One doctor expressed her view that smoking was a negative health decision, she thought a lack of commitment to smoking cessation was related to people with COPD not wanting to take ownership of their health and adhere to pulmonary rehabilitation:

‘if they’re not engaged in doing things for themselves, for example like stopping smoking, they will be more unlikely to be engaged with a programme where they have to take responsibility.’ (DR 1, 25–30, H1)

There was also the view that ‘*compliance is a big issue with COPD patients*’ (DR 5, 25–30, H2) and that they lacked conformity and would not engage with health-related programmes. These illness beliefs defined their perceived suitability for pulmonary rehabilitation.

### Pulmonary rehabilitation beliefs – primary care

All healthcare professionals in primary care discussed opinions of what pulmonary rehabilitation entails and patient suitability. Several GPs provided a general definition of pulmonary rehabilitation, rather than focussing on details of their local programme. They often concentrated on the exercise component describing it as ‘*an exercise programme used to improve the breathing’* (GP 4, 31–40). Although, others appeared unsure and speculated what their local programme entailed:

‘I think it's quite limited [knowledge] I’m afraid, I just know that you can refer into it and then the patients have like, probably about, I think a set number of sessions, like six or eight sessions in a programme.’ (GP 2, 31–40).

Practice nurses recognised a purpose of pulmonary rehabilitation was providing an escape ‘*coffee and a chat*’ (PN 3, 51–60) for those who may be socially isolated. They viewed pulmonary rehabilitation as increasing health-related knowledge, and improving well-being:

‘Pulmonary rehabilitation has been providing a source of education with supportive data demonstrating positive outcomes on quality of life for years’ (PN 3, 51–60)

Perceived patient suitability was commonly discussed, and healthcare professionals held different criteria as to who they would consider for pulmonary rehabilitation. Some appeared to follow general guidance set by the local programme suggesting ‘*they have to have roughly an MRC [Medical Research Council Dyspnoea Scale] 3 scale’* (PN 3, 51–60). Others believed it may not be suitable for those who were continuing with daily activities and able to work. An internal conflict was apparent, as it was deemed referral should not be left too late:

‘there are patients that are still active and working, and coping with COPD, and it might not be suitable for them because they’re not actually experiencing any difficulties. But then we’ve got to be careful that we don’t wait till they are experiencing very, very vast difficulties.’ (PN 2, 41–50)

Frequent exacerbations were a prominent symptom which triggered GPs to consider pulmonary rehabilitation, as it was often deemed appropriate for those at the worse end of the spectrum. Strikingly, several GPs considered pulmonary rehabilitation as a last resort:

‘I see quite a lot of people who are on maximal therapy and have multiple exacerbations of their COPD, I could refer some of those I suppose’ (GP 4, 31–40)

Others referred patients for the psychological support provided, rather than any overt physical benefits. They felt strongly about the depth of psychological impact associated with COPD, which included refrainment from activities and a considerable impact on quality of life. For those impacted in this way pulmonary rehabilitation was viewed as a lifeline, and a prompt to start discussions about the programme:

‘I might be talking with someone about you know, when they were going out and seeing their friends … the things that make us smile and bother to stay on this earth sort of thing, and if their breathing was something that was stopping them doing that, or their confidence in their breathing was something that was stopping that, then I might start a conversation [about PR]’ (GP 3, 41–50)

There was a disparity between perceived patient suitability with some aware of and adhering to local programme criteria, and others making their own presumptions based upon individual patient characteristics.

### Pulmonary rehabilitation beliefs – secondary care

Opinions ranged regarding patient suitability and the characteristics required to attend pulmonary rehabilitation. Doctors perceived pulmonary rehabilitation was for patients with *‘worsening breathlessness, their inhalers aren’t really working, you’ve kind of tried everything*’ (DR 1, 25–30, H1), whereas general nurses believed it was better if ‘*you catch them early, to start them off with these exercises*’ (GN 4, 41–50, H2).

A notable finding in secondary care was many ‘*didn’t really know it [PR] was a service’* (DR 2, 25–30, H1) or what it involved. Each healthcare professional in secondary care worked on a general medical ward with many COPD patients; a prominent feature of many interviews was: ‘*it's the first time I’ve heard about it* [PR]’ (DR 3, 31–40, H1). Although not intentional, participation in the research interview raised awareness for many.

The term *rehabilitation* caused confusion, with it sometimes being associated with a bridge between hospital and independent living. One general nurse highlighted uncertainty when they asked: ‘*are you talking about when they’re going home?*’ (GN 4, 41–50, H2). Rehabilitation was associated with the perception that patients can return to a state of health held prior to a diagnosis or accident. This was not deemed possible for those with COPD and therefore the name ‘pulmonary rehabilitation’ resulted in confusion:

‘we don’t really have anyone on that, the rehab ward, I have worked in the rehab ward, that was for hips.’ (GN 5, 51–60, H2)

Interestingly, many were aware of cardiac rehabilitation and chose to discuss this instead. One general nurse who had been working with COPD patients for many years was shocked:

‘I’ve worked in acute medicine for 20 years, and I’ve never heard of pulmonary rehab, I’ve heard cardiac rehab but I’ve not heard pulmonary rehab.’ (GN 2, 41–50, H1)

Despite lacking knowledge of pulmonary rehabilitation, healthcare professionals discussed how they could appreciate the potential benefits. The non-medicalised approach was considered to improve lung capacity and ultimately enhance patients’ ‘*well-being and way of life*’ (DR 1, 25–30, H1). Healthcare professionals valued the educational aspect, as this was deemed vital in assisting self-management of symptoms. Education was perceived to provide a pragmatic, representational view of disease progression:

‘it can educate them, so they can try and pre-empt and reduce exacerbations, and try and find ways in which they can manage their condition a lot better.’ (GN 7, 41–50, H2)

### Organisational and referral pathway perceptions – primary care

Many in primary care deferred the responsibility of referral. This appeared closely associated with not considering pulmonary rehabilitation a priority. Some GPs discussed how they previously made referrals but now felt deskilled. They felt it was not their role to refer patients, and often deferred responsibility to practice nurses:

'She's [nurse practitioner] basically as good as a GP… but she's got a lot more knowledge and confidence to manage this [pulmonary rehabilitation], so she can admit to hospital and all sorts of things’ (GP 8, 51–60)

Others ‘*kind of assumed that they’ve [patients] already been referred’* (GP 4, 31–40), and regarded it as the role of secondary care, or should be ‘*automatic*’ (GP 5, 41–50), after a hospital admission. Interestingly, after a period of reflection one GP came to the realisation that other healthcare professionals may also assume someone else is initiating referral:

‘I bet quite a few of the secondary care [healthcare professionals] think that we’re doing it all [referrals]’ (GP 8, 51–60)

Practice nurses took greater responsibility for referrals and when discussing pulmonary rehabilitation were predominantly positive. They however felt too much pressure to complete a referral during a COPD annual review. Although not deemed the most appropriate decision on some occasions due to time restrictions, pulmonary rehabilitation was sometimes overlooked:

‘due to time constraints and being busy I just thought, oh I’ll wait and we’ll review you next year and see how you’ve got on, but really I felt that the intervention would have been better early.’ (PN 2, 41–50)

There was agreement that better communication was required between the service and referrers. It was considered difficult to be knowledgeable about the benefits of pulmonary rehabilitation:

'So, there's no real details about the course itself … we never got this is week one, week two, week three, the content … we were referring people blind if you like.’ (PN 1, 61+)

Practice nurses believed referrals took tremendous effort but were disheartened that the pulmonary rehabilitation team did not inform them of patient progress. It was perceived their efforts would be worthwhile if they knew the patients had benefitted from the sessions. There was the notion that a lot was expected from them, however they received very little back from the service:

‘it seems like a one way road of information, that we send loads of information about medication, spirometry, history, all this sort of stuff, and we either get patient attended pulmonary rehab, or patient failed to attend’ (PN 1, 61+)

Uncertainty was coupled with the perception pulmonary rehabilitation was a complicated sell. Many found it difficult to initiate conversations surrounding pulmonary rehabilitation, as they perceived ‘*a lot of them [patients] are not interested to start with’* (GP 5, 41–50). Practice nurses tried to encourage attendance, although this was not always straightforward; it was considered the phrase ‘*light exercise*’ (PN 1, 61+) evoked fear. Others believed the ease of the sell was dependent on individual demographics:

‘I’ve come to a much younger group, I mean the number of people in their forties and fifties that we’re diagnosing, got quite a high cannabis use area as well, and basically just to get them to understand that something that isn’t a medicine is going to help them, is a much more difficult concept to get across to this group’ (PN 3, 51–60)

Referrals were considered time-consuming, and in some cases acted as a deterrent. There was the notion that: ‘*if referrals are made easy then you do a lot more of them*’ (PN 2, 41–50). It was evident key facilitators, such as having a ‘*simple pro-forma*’ (GP 2, 31–40) greatly assists referral.

### Organisational and referral pathway perceptions – secondary care

All healthcare professionals in secondary care believed there was a lack of awareness and publicity surrounding pulmonary rehabilitation. Low awareness was often attributed to pulmonary rehabilitation not being discussed in detail, or forming a core part of the COPD curriculum during either medical or nursing degree:

‘it's just mentioned as a part of the management plans generally in terms of medical education. There's no formal mention of it [pulmonary rehabilitation] really’ (DR 4, 25–30, H1)

Lack of publicity was closely aligned with a lack of awareness*,* however in this instance healthcare professionals often blamed the service for their dearth of knowledge. It was considered the responsibility of the service to raise awareness and the profile of pulmonary rehabilitation:

‘I’ve known nothing about it before, so if there are services available it probably does need to be advertised a bit more.’ (DR 5, 25–30, H2)

Many in secondary care also deferred responsibility to justify their lack of referral. They considered it was not their role as they ‘*don’t deal with that* [pulmonary rehabilitation]’ (GN 5, 51–60, H2). A prominent justification was the primary role of those working on general medical wards was to assist patients with their acute condition. Hospital was considered as a holding area, where medical intervention was administered. The responsibility of referral was deferred to primary care:

‘in hospital, especially in the acute wards, we’re so focused on getting people into hospital, treating them for their acute conditions and then sending them home when they are back to their baseline. So, I think therefore there's probably more of a place in primary care, in terms of it being a bit more of a holistic approach to, to their treatment.’ (DR 1, 25–30, H1)

Although not specifically articulating a deferral of responsibility, a doctor contemplated the situation in primary care:

‘I don’t know whether the GPs have better knowledge of it, or whether it's just hospital doctors that don’t’ (DR 5, 25–30, H2)

Healthcare professionals in secondary care also discussed patient suitability and how to refer as a prominent issue. Being unsure of patient eligibility and the referral process acted as a barrier. Some stated ‘*I wasn’t aware I could [refer to pulmonary rehabilitation]’* (GN 6, 41–50, H2). Many also stated that they ‘*don’t know what the requirements are*’ (DR 1, 25–30, H1).

Overall, it was apparent that many clinicians on general medical wards would ‘*definitely consider*’ (DR 1, 25–30, H1) referral to pulmonary rehabilitation if aware of patient suitability and if the referral process was simple and undemanding.

## Discussion

This study is the first to investigate the perceptions of healthcare professionals working in primary care and on general medical wards within secondary care, regarding pulmonary rehabilitation as a management strategy for individuals with COPD. The findings raise several important considerations surrounding healthcare professionals’ perceptions of pulmonary rehabilitation and the ability for these beliefs to impact referral practice. It was surprising that, healthcare professionals (aware of programmes such as cardiac rehabilitation) largely lacked knowledge of pulmonary rehabilitation. Poignantly, none of those working on General medical wards had referred a COPD patient to pulmonary rehabilitation, despite caring for those with COPD daily. This study identifies a need for enhanced awareness of non-pharmacological management strategies to support those with COPD, with many healthcare professionals favouring a medicalised approach.

The associated beliefs surrounding a diagnosis are referred to as illness perceptions; these are internal beliefs which individuals develop to try to make sense of their condition.^21^ Healthcare professionals illness perceptions regarding COPD were surprisingly varied, with some holding negative views surrounding the cause of COPD, for example considering it as a smoker's disease. Others generalised COPD patients’ motivation surrounding exercise programmes and held opinions surrounding their socio-economic status and education. This supports Mathiopudakis et al.'s work which highlights a stigmatisation that some individuals with COPD feel from healthcare professionals and the impact this has on the care they receive.^22^ Previously, the literature has focussed upon COPD illness perceptions from a patient's view with regards to how they perceive, and then manage their condition,^23^ the current study extends this to investigate beliefs from a healthcare professionals’ perspective.

Within primary care healthcare professionals focussed on the psychological impact of COPD, such significance, however, was not placed on physical symptoms. In contrast, in secondary care the complexities of the physical symptoms were centred upon, and no cure. The burden of multiple symptoms and the impact on quality of life has been previously discussed.^4^ It could be proposed the strict use of the medical model may influence healthcare professionals’ perceptions of the disease, whereby focus is placed upon treating the condition rather than assisting patients to live with it. In the current study, in secondary care it was perceived that exercise induces breathlessness. This illness perception has the potential to impact referral to pulmonary rehabilitation and is consistent with a Spanish study of 338 medical students (47.1%), who did not recommend exercise for those with COPD.^24^

Negative beliefs surrounding COPD were common in primary and secondary care. Despite evidence to display the correlation between the incidence of COPD and smoking,^25^ the frequency with which healthcare professionals in the current study mentioned smoking, suggests they consider COPD as a smoker's disease. The findings of the current study highlight smoking being associated with receipt of benefits and geographical location. Notably, it appeared some healthcare professionals stigmatised whole communities, or groups, based upon the cognitive representation they had built regarding their practice location; something not previously discussed in the literature with regards to COPD. Reference to compliance, concordance, and adherence to self-management has however previously been referred to.^26^ The literature also emphasises that socio-economic status and where an individual lives can also impact adherence to pulmonary rehabilitation.^11^ The findings of the current study highlight those in secondary care perceive COPD patients as being unable to take control of their health, are resistant to change, and this was related to the notion patients often lacked compliance and engagement with healthcare.

Unfamiliarity with pulmonary rehabilitation eligibility criteria has previously been discussed within the literature.^15^ Fundamentally, healthcare professionals in primary care in the current study had a good understanding of what pulmonary rehabilitation entails, yet some questioned the ideal time to refer and whether this should be pre or post significant impact upon lifestyle. Those in secondary care in the current study perceived pulmonary rehabilitation as an add on. Others perceived eligibility would depend upon the age of the patient, believing younger patients would achieve more. This however is a misconception and a lack of belief in older patients’ abilities, as pulmonary rehabilitation can improve lung function, general health, and quality of life in the elderly.^27^

The effectiveness of pulmonary rehabilitation in reducing COPD related hospital admissions is proven^4^ and given the concern from healthcare professionals in secondary care surrounding pressures on hospital capacity, the lack of awareness surrounding pulmonary rehabilitation was an unexpected finding. Healthcare professionals never having heard of pulmonary rehabilitation has been highlighted previously in Saudi Arabia,^28^ however this is not a known finding in the United Kingdom. There were commonalities in perceptions of barriers to the programme between professional groups and primary and secondary care, such as location, transportation and session times. These issues are prominent within the literature, for healthcare professionals,^28^ and patients alike.^6^

In the current study, healthcare professionals on general medical wards attributed their lack of awareness of pulmonary rehabilitation to limited publicity. Consistently, pulmonary rehabilitation was viewed as being missing from the medical and nursing curricula. This is a unique finding, as it is believed a lack of teaching has not previously been attributed as a barrier to referral.

Deferral of responsibility was discussed in primary and secondary care, and the pertinence of this issue is reinforced in the literature.^15^ The lack of responsibility for referral contradicts the patient centred care approach, which endeavours to meet patient needs whilst providing a positive healthcare experience.^29^ In the current study, it was unusual for practice nurses to defer referral responsibility. Referral often took place during a 30-minute COPD annual review; practice nurses believed there was not enough time to complete other tasks in addition to referral. Restricted primary care appointment times have also been cited as a barrier in Australia,^30^ highlighting the issue is not exclusive to the UK. In the current study, other than practice nurses, everyone deferred referral, each believing it was another's role. This could also further emphasise the lack of referrals highlighted in the National Pulmonary Rehabilitation Audit.^18^

This is the first study to include the perceptions of both those working in primary care and secondary care in relation to pulmonary rehabilitation for those with COPD. Attention was given to the suitability of IPA as it could be argued 27 is a large sample size. It is however suggested there is no definitive answer to the correct number of participants in IPA, as data collection should be driven by the richness of the data obtained and dependent upon how the researcher compares different accounts.^20^

A potential weakness was that interviews were conducted by a non-healthcare professional (ES), however this instead appeared to facilitate conversation, with healthcare professionals providing examples with no assumption of shared knowledge, resulting in rich data being collected. Challenges associated with assumed knowledge and power dynamics have previously been highlighted, and it is suggested that reflective notes can mitigate these issues,^31^ something which the current study employed.

In conclusion, referral to pulmonary rehabilitation appears dependent upon individual healthcare professionals, their perceptions of pulmonary rehabilitation, how COPD affects patients, and their knowledge of referral. These aspects pieced together, could act as a predictor of referral practice and inform strategies for increasing referral rates. It is hoped this research will contribute to the limited knowledge of healthcare professionals’ perceptions of pulmonary rehabilitation and add a unique perspective and potential explanation as to why the National Pulmonary Rehabilitation Audit^18^ concluded referrals to pulmonary rehabilitation were lacking. Further research would be beneficial to survey those working in primary care and secondary care across the UK, to assess if the findings could be generalised, and highlight beliefs with regards to pulmonary rehabilitation amongst a larger group. Those working on respiratory wards would add further insight, to determine if their knowledge or understanding of the programme is also lower than expected and how often they refer. Lastly, given healthcare professionals discussed a lack of teaching or exposure to pulmonary rehabilitation during their medical or nursing degrees, additional research is required to determine how pulmonary rehabilitation is incorporated into the curricula.

## Clinical messages

- The illness perceptions healthcare professionals hold about chronic obstructive pulmonary disease can influence pulmonary rehabilitation referrals.
- Greater communication is required between healthcare professionals surrounding referral to pulmonary rehabilitation. It should not be assumed other colleagues are making a referral.
- Better education and awareness raising is required for healthcare professionals. This should come from local pulmonary rehabilitation services and be better integrated into medical and nursing degrees.
